# Global Citizenship: Buzzword or New Instrument for Educational Change?

**DOI:** 10.5964/ejop.v15i2.1999

**Published:** 2019-06-07

**Authors:** Abdeljalil Akkari, Kathrine Maleq

**Affiliations:** a University of Geneva, Geneva, Switzerland

Worldwide, deep societal changes brought by globalization, the expansion of ICT (information and communication technology) and increased cultural and ethnic diversity have entailed an unprecedented rise in interest for post-national and global citizenship models. This has resulted in a growing body of literature from various fields such as education, psychology and philosophy. However, global citizenship seems to be both an attractive and contested concept. Attractive because we all seek to find answers as to how to better live together in a globalized world. Contested because it appears conceptually fragile and difficult to implement in national contexts (Dower & Williams, 2016; Pashby, 2015). In the first part of this editorial, we analyze how it has become a strong focus in educational, political and intellectual discourse. The second part addresses the lack of conceptual clarity as well as the conceptual divides associated with it. The third part describes the possibilities and opportunities of implementing global citizenship in educational landscapes.

## International Organizations: Initiatives and Deadlocks

The concept of “citizen of the world” is not a new idea and can be traced back to cosmopolitan cities that have produced philosophers, writers, artists, and thinkers able to see their identity across national, cultural and linguistic boundaries. Two decades ago, international organizations gave a new impulse to the utopian concept of global citizenship by making it a crucial line of action, aimed at addressing the challenges of the 21th century linked to globalization, crisis of traditional conceptions of civic and citizenship education and global environmental issues.

Firstly, globalization is mostly driven by economics, business and technology, creating a continuous flow of products, capital, people and information across the world. Although linked to globalization, the concept of global citizenship refers to a shared sense of identity and human values. While globalization is under political debate, we need, more than ever, to form global citizens. Indeed, even if globalization has opened for some regions in the world access to a global market and technological advances (Guibernau, 2010), it has resulted in major environmental and social challenges.

Secondly, traditional conceptions of civic and citizenship education in many nations-states have failed to meet the challenges of multiculturalism. In many countries encountering immigration, the political climate is marked by a binary divide between “those who assert that in a globalized world and nation-states characterized by diversity, one requires a primary commitment to the nation-state” (Osler, 2011, p. 1) and those who emphasize diversity and inclusion. In this respect, Banks et al. (2005, p. 25) calls appropriately to “find ways to delicately balance unity and diversity”. Cultural diversity is equally a current issue in contexts where “globalization is contributing to the expansion of certain values, ideologies and products resulting in a pervasive, if uneven, cultural and linguistic homogenization characterized by US and Western influence” (Guibernau, 2007, p. 140). This has resulted in a genuine concern from a number of nations and ethnic groups about cultural and linguistic preservation (Guibernau, 2010).

Thirdly, environmental issues and climate change transcends national borders and have global consequences. These issues clearly underline our interconnectedness and the need for collective and political action on a global level. Environmental awareness is therefore an essential attribute of global citizens.

In response to these challenges and following the Secretary‐General Ban Ki‐moon UN Global Education First initiative in 2002 which placed the promotion of global citizenship as one of its top three priorities, international organizations have brought the concept of global citizenship under the spotlight. The Education 2030 Framework for Action, within the UN 2030 Agenda for Sustainable Development adopted in September 2015, stresses the crucial role of education in promoting democracy and human rights and enhancing global citizenship, tolerance and civic engagement as well as sustainable development (Robiolle Moul, 2017). Since then, the idea has attracted more attention from the international community and an unprecedented rise in interest from scholars (Robiolle Moul, 2017). From then on, global citizenship has become the new buzzword in educational landscapes around the world.

However, this increased focus on global citizenship is hampered by anti-globalization rhetoric, growing skepticism towards multiculturalism, a rise of nationalism and anti-refugee discourse. Moreover, the ineffectiveness and the relative failure of international organizations in global governance raise doubts about the concept of global citizenship. The United Nations system was created to peacefully resolve conflicts between states and to ensure stability and world peace. Reality demonstrates that the persistence of armed conflicts and the withdrawal of some powerful States from international organizations and international covenants diminishes international governance. Even global economic governance is currently under threat due to trade wars between different countries and economic spaces.

The need for learners to build an understanding of global issues and become responsible and active global citizens is therefore stronger than ever. Efforts to achieve sustainable development will require finding a delicate balance between economic, environmental and social goals. In this respect, Davies et al. (2018, p. xxv) suggest that “global citizenship education is critical for achieving sustainable development, especially as both areas struggle to find a place in the school curriculum”.

## Neoliberal, Radical, and Critical Approaches to Global Citizenship

Differing conceptions of global citizenship have resulted in ongoing disagreements about its definition. Aktas, Pitts, Richards, and Silova (2017) distinguish three main approaches to global citizenship: neoliberal, radical, and critical.

In the neoliberal approach, the focus is on developing “global competencies” that would enable students to become internationally mobile and readily employable in a variety of cultural and national contexts. The goal of global citizenship within the neoliberal framework is to facilitate the integration of individuals and nations in the global marketplace (Aktas et al., 2017; Rizvi, 2007). The key idea being that individuals should be able to move freely throughout the global world regardless of national borders in order “to increase transnational mobility of knowledge and skills with the goal of linking global citizenship directly to global economic participation“ (Shultz, 2007, p. 252).

The radical approach differs from the mainstream, normative and “civilizing” neoliberal approach of global citizenship, recognizing the existence of global power dynamics and inequalities (Andreotti, 2006). The radical perspective of global citizenship adopts a critical stance on global structures that serve to perpetuate global inequalities and deepen the North-South divide (Shultz, 2007). Within this approach, the role of the global citizen is to challenge the hegemony of economic globalization and build solidarity across marginalized groups to fight oppression rather than focusing on building economic relationships across the globe (Aktas et al., 2017).

The critical approach to global citizenship calls for a transformation of not only institutions and systems but also personal and cultural mind-sets. Furthermore, this approach stresses the need to provide opportunities for reflexive learning and critical thinking, allowing students to become active and responsible citizens. Through exposure to situations with different cultures and groups, the critical approach focuses on individual responsibility for social change (Aktas et al., 2017; Boni & Calabuig, 2015). This approach is in line with Torres’s (2009) work that advocates for critical social and political perspectives in citizenship. In the educational context, critical approaches to global citizenship aim to shift the focus of democratic citizenship discourse away from the symbolic (legal and political rights taught through civic education) to emphasize active participation and action towards social equality, justice and freedom (Isaacs, 2018).

Furthermore, it is important to highlight the main conceptual weakness. Global citizenship is often regarded as merely a theoretical concept compared to national citizenship, since the global society still lacks key aspects of polity such as rule of law, democracy, representativeness, and accountability (Chung & Park, 2016). Moreover, Davies et al. (2018) suggest that global citizenship could be considered an ‘empty signifier’ that different concepts, perspectives and ideologies attempt to ‘fill’ with meaning. In the context of education, the ‘emptiness’ of the concept offers multiple possibilities for democratic practices mainly in informal settings and extracurricular and community projects. However, it may in turn represent a challenge to educators and policy-makers attempting to grapple with how to bring global citizenship education into pedagogical practice.

Finally, to observe globalization as a self-evident good is to avoid confronting the fact that globalization affects differently individuals, groups and nations. Thus, contexts such as geographical positioning (the global North versus the global South) and, historical legacy (slavery, colonialism) situate people and nations contextually in terms of national culture, political organization and economic activity. Consequently, from an education policy perspective, it would seem that particular (national) citizenship education policies must assign priority to their local needs and aspirations before any thought is given to global citizenship education (Isaacs, 2018; Mannion et al., 2011).

## Rethinking Education by Implementing Global Citizenship

Worldwide, we are witnessing an historic shift in identity models and sense of belonging. Increasingly, students are less tied to specific location, social structure, or nation-state. Social networks are borderless and globalization has gone digital. Smartphones and other mobile devices give us an unprecedented level of global interconnectedness which we are broadly speaking not prepared for in society and educational institutions. Reimers (2006, p. 277) rightly stated that “globalization is one of the most important changes taking place in societies around the world today and yet it is unclear that schools have realigned their purposes to prepare their students to be competent citizens in an age of globalization“.

Individual do not have an innate understanding of our shared humanity but learn this over time through socialization, education and schooling. Global citizenship is therefore fostered through education. Thus, the production of a global citizen is a shared responsibility between society and educational systems.

It is, however, important to distinguish between what the educational system can do in primary, secondary and tertiary educational sectors. At primary and secondary levels, it appears challenging to integrate education for global citizenship. On the one hand, nation-states are anxious to control and standardize school curricula. On the other hand, the timetable of schooling is under pressure from all sides with many requests for the introduction of new content: ICT, entrepreneurship education, health education, civic education, etc.

A recent comparative UNESCO’s study suggested conceptualizing the many differences and similarities regarding global citizenship in official curricular prescriptions within three categories. The first category *affirms and develops* the concept of global citizenship education and associated ideas or topics as constituent components of citizenship education. The second category *recognizes* the concept and associated ideas or topics and their relevance to citizenship education, without consistently integrating them into the curriculum. The third category *ignores* the concept and associated ideas or topics, not mentioning them as part of the citizenship education area in the curriculum. The countries of the study distribute themselves more or less evenly among these three positions. The South Korean and the Indonesian curricula fall clearly within the first category, affirming and consistently developing the concept in their prescriptions. The curricula in Costa Rica, Colombia and Iraq fall under the second category: all three of them recognized the concept and included it in their definitions, but not consistently. Finally, the curricula in England, France and Kenya are in the third category, ignoring the concept of GCE defined by UNESCO (Cox, 2017). However, this does not mean that global dimensions are not addressed in the curricula.

Universities have however been, up until how, preserved from the hyper-standardization of curricula since they still have academic freedom and room to design interesting and innovative programs. Nevertheless, today we need to rethink higher education in a global age. New technologies offer new possibilities for learning and student cognitive development.

Since today’s students tend to see the world and themselves as more global, borderless and fluid, and a high percentage of students in the world’s biggest cities are from immigrant backgrounds, global citizenship could provide an opportunity to innovate our teaching, pedagogies, methodologies and instruments in higher education.

## Conclusion

This article draws attention to the concept of global citizenship as, potentially, an instrument of educational change in increasingly diverse societies. However, in order to go beyond a simplistic approach which limits itself to adding international content or token global education type activities to citizenship education programs, global citizenship education ought to emphasize active participation and action towards social justice and sustainability (Akkari & Maleq, 2019). Learning global citizenship may only be accomplished through solving complex problems that require interdisciplinary collaboration, great creativity and close collaboration among students, teachers, and other stakeholders. In this respect, informal education offers some potential for global citizenship projects. Indeed, the flexibility within informal education allows learners to engage with communities on a local and global scale.
